# Die Geschichte des implantierbaren Kardioverter-Defibrillators in Deutschland

**DOI:** 10.1007/s00399-024-01001-5

**Published:** 2024-02-29

**Authors:** Michael Block, Helmut U. Klein

**Affiliations:** 1 Gräfelfing, Deutschland; 2 Hannover, Deutschland

**Keywords:** Implantierbare Elektroden, Plötzlicher Herztod, Ventrikuläre Tachykardie, Kammerflimmern, Medizingeschichte, Implantable electrodes, Sudden cardiac death, Ventricular tachycardia, Ventricular fibrillation, History of medicine

## Abstract

Der implantierbare Kardioverter-Defibrillator (ICD) war ein Durchbruch in der Prävention des plötzlichen Herztodes. Nach jahrelanger technischer Entwicklung durch Michel Mirowski erfolgten trotz vieler Widerstände in den USA 1980 erste Implantationen. Es konnte gezeigt werden, dass Kammerflimmern zuverlässig durch den ICD automatisch erkannt und defibrilliert wurde. Knapp 4 Jahre nach den USA begannen mehrere Zentren, in Deutschland ICDs zu implantieren. Bald wurden außerhalb der USA in Deutschland die meisten Patienten versorgt. Die absolute Zahl der Implantationen war weiterhin klein, solange nur Implantationen mittels Thorakotomie und epikardialen Defibrillationselektroden möglich waren. Anfang der 1990er Jahre konnte ein ICD pektoral, wie ein Schrittmacher, in Kombination mit einer transvenösen Defibrillationselektrode implantiert werden. Die Implantationszahlen stiegen sprunghaft an. Die klinische Forschung in Deutschland begleitete die technischen Fortentwicklungen und hier erfolgten viele First-in-human-Studien. In den USA und Deutschland wurden 1991 erste Leitlinien zur Indikation herausgegeben. Randomisierte Studien zur Mortalität, zumeist unter amerikanischer Leitung mit deutscher Beteiligung, aber auch unter deutscher Leitung (CASH, CAT, DINAMIT, IRIS), wurden zwischen 1996 und 2009 publiziert. Die Ergebnisse dieser lang zurückliegenden Studien wurden 2016 in Frage gestellt, als die DANISH-Studie keine signifikante Verbesserung der Mortalität ergab. Die Implantationszahlen sind seitdem rückläufig. Derzeit wird daran geforscht, genau die Patienten zu versorgen, die trotz optimaler Therapie vor dem plötzlichen Herztod geschützt werden müssen. Risikoscores unter Einbeziehung myokardialer Narben in der Magnetresonanztomographie (MRT) und genetischer Information sollen hierzu beitragen.

## Abstract (en)

The implantable cardioverter–defibrillator (ICD) was a breakthrough in the prevention of sudden cardiac death. After years of technical development in the USA, Michel Mirowski succeeded in proving reliable automatic defibrillation of ventricular tachyarrhythmias through initial human implantations in 1980, despite many obstacles. Nearly 4 years later, the first patients received ICDs at multiple centers in Germany. Subsequently, outside the USA, Germany became the country with highest implantation rates. The absolute number of implantations remained small as long as implantations required epicardial defibrillation electrodes and therefore thoracotomy by cardiac surgeons. Pacemaker-like implantation using a transvenous defibrillation electrode with a pectoral ICD became feasible in the early 1990s pushing implantation rates to the next level. Technical advancements were accompanied by clinical research in Germany, and often, the first-in-human studies were conducted in Germany. In 1991, the first guidelines for indications were established in the USA and Germany. Several randomized studies on indications were published between 1996 and 2009, mostly led by American teams with German participation, but also under German leadership (CASH, CAT, DINAMIT, IRIS). The DANISH study in 2016 questioned the results of these long-standing studies. Instead of providing ICDs to patients using a broad indication, future efforts aim to identify patients who, despite optimal medical therapy, cardiac resynchronization therapy (CRT), and/or catheter ablation, need protection against sudden cardiac death. Risk scores incorporating myocardial scars in magnetic resonance imaging (MRI) and genetic information are expected to contribute to more individualized and effective indications.

## Die ersten implantierbaren Defibrillatoren in Deutschland

**Figure 1:**
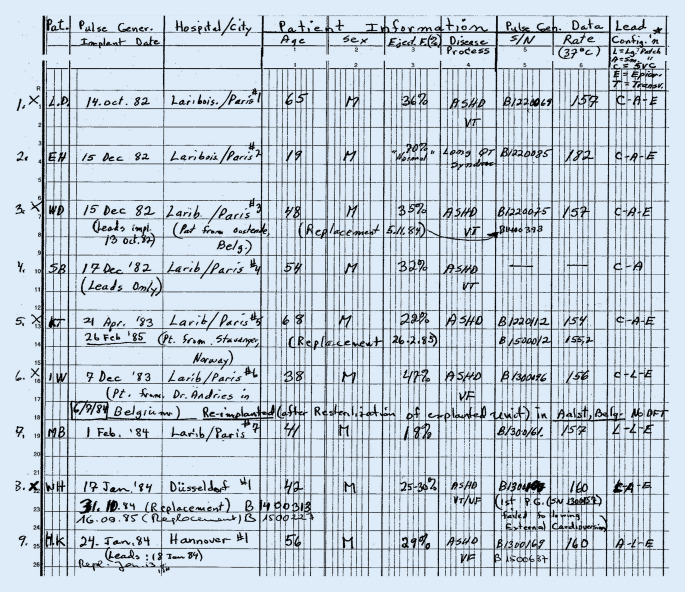

**Figure 2:**
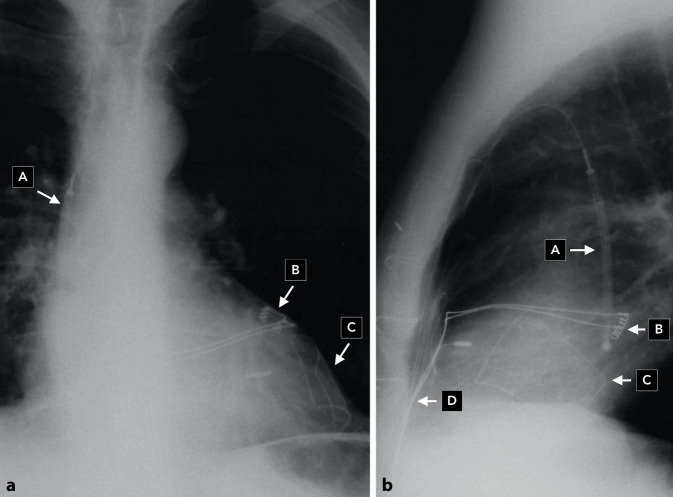

**Figure 3:**
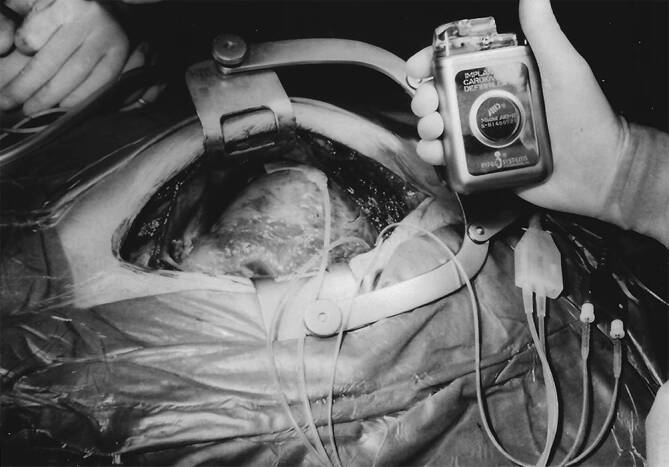

Erstmalig wurde am 04.02.1980 am Johns Hopkins Hospital in Baltimore ein automatischer Defibrillator (AID) der amerikanischen Firma Medrad/Intec Systems, Pittsburgh, Pennsylvania, USA implantiert [1], und am 07.10.1980 erschien im *New England Journal of Medicine* die Veröffentlichung über die ersten 3 Implantationen in den USA [2]. Die ersten publizierten AID-Patienten hatten vor der Defibrillator-Implantation zahlreiche Ereignisse mit ventrikulären Tachykardien (VT) und Kammerflimmern trotz Verwendung multipler Antiarrhythmika. Erstautor war der Kardiologe Dr. Michel Mirowski, der Erfinder des implantierbaren Defibrillators. Er hatte weit über 10 Jahre gebraucht, um sein Projekt zu realisieren, und musste bis zur ersten Implantation viele Widerstände überwinden und Kritik ertragen [3]. Hier zu nennen ist auch, dass sein Projekt von einer großen Schrittmacherfirma intern gestoppt wurde und der Nobelpreisträger Bernard Lown sich 1972 in einem Editorial in *Circulation* gegen die Entwicklung eines implantierbaren Defibrillators aussprach [4]. Da Mirowski als polnischer Jude mit 15 Jahren aus Warschau 1939 nach Russland vor den Nazis floh, aber seine Familie und die meisten seiner Freunde und Mitschüler den Holocaust nicht überlebten, genehmigte Mirowski Defibrillator-Implantationen in Deutschland erst 1984, nachdem bereits seit 1982 sechs Patienten von Philippe Coumel einen implantierbaren Defibrillator in Paris erhalten hatten (Abb. 1). In Frankreich hatte Mirowski nach dem Krieg Medizin studieren können, bevor er Europa nach Israel verließ, später zunächst in Mexiko mit Sodi-Pallares und Cabrera arbeitete, dann bei Helen Taussig in den USA forschte und schließlich seit 1968 als Medical Doctor am Sinai Hospital in Baltimore, einem Teaching Hospital der Johns Hopkins Universität Baltimore zusammen mit Morton Mower am AID arbeitete [5]. Am 18.01.1984 erfolgte die erste Implantation eines Defibrillators in Deutschland in der Herzchirurgie der Universitätsklinik Düsseldorf (Wolfgang Bircks, Jörg Ostermeyer, G. Breithardt) im Rahmen einer Bypass-Operation [6]. Eigentlich hätte die deutsche Erstimplantation am Vortag an der Medizinischen Hochschule in Hannover erfolgen sollen, konnte auf Grund eines Gerätedefektes dort aber erst eine Woche später stattfinden. Es handelte sich bereits um die 2. Generation des implantierbaren Defibrillators (AID-B), der auf Grund der Verwendung von zwei epimyokardialen Schraubelektroden nicht nur Kammerflimmern, sondern auch VT detektieren, und auch kardiovertieren konnte. Die Schockabgabe erfolgte zunächst bei den ersten 7 Implantationen in Europa – wie auch in Düsseldorf – mit einer Defibrillationselektrode in der Vena cava superior und einer großen Patch-Elektrode auf dem Epikard des linken Ventrikels (Abb. 2). Bei der ersten AID-B-Implantation in Hannover wurden dann erstmals zwei epikardiale Patch-Elektroden (eine große und eine kleinere) zur Defibrillation verwendet (Abb. 3). Das machte die zusätzliche Implantation der Vena-cava-superior-Elektrode überflüssig. Dieses Verfahren wurde für die nächsten Jahre dann das Standard-Implantationsverfahren. Anstatt der „probability-density-function“ zur Detektion von Kammerflimmern gab es nun ein Frequenzkriterium, das über die Durchführung der Kardioversion/Defibrillation entschied, welches werkseitig fix programmiert war; für die ersten beiden deutschen Patienten bei 160/min. Das Aggregat wurde in die vordere Bauchwand subkutan oder unter den linken M. rectus implantiert. Ein Jahr später, 1985, wurde Intec Systems von Cardiac Pacemakers Inc. (CPI), Saint Paul, Minnesota, USA gekauft, und es erfolgte die Marktzulassung des ICD in den USA durch die Food and Drug Administration. Diese von der Fa. CPI hergestellten und auch in Deutschland von vielen Zentren implantierten ICDs hatten das Trademark „AICD™“.

## Entwicklung des transvenösen Defibrillationssystems

Der Durchbruch bei der Verbreitung des implantierbaren Kardioverter-Defibrillators erfolgte aber erst nach dem Ersatz der zwei epikardialen Patch-Defibrillationselektroden durch eine transvenöse rechtsventrikuläre Sense/Pace/Defibrillationselektrode und eine subkutane Patch-Elektrode am linkslateralen Thorax, was nun keine Thorakotomie mehr erforderte. Ein in den USA bereits 1987 erstmalig implantiertes transvenös/subkutanes System der Firma CPI [7] musste wegen Elektrodenbrüchen der Endotak™-Elektrode zunächst überarbeitet werden [8]. In Europa wurde dann in Münster am 10.10.1989 erstmalig das transvenöse/subkutane PCD™-System (Trademark, „Pacemaker Cardioverter/Defibrillator“) der Firma Medtronic, Minneapolis, Minnesota, USA, mit der Transvene™-Elektrode implantiert [9]. Die überarbeitete Endotak™-Elektrode wurde ab Mai 1990 in Europa und ab September 1990 in USA in Studien untersucht. Das Erreichen einer zuverlässigen Defibrillation war bei einem größeren Anteil der Patienten trotz verschiedener Elektrodenkonfigurationen mit den verfügbaren monophasischen Schocks nicht möglich [10]. In einer im Mai 1991 in Münster begonnenen Cross-over-Studie konnte gezeigt werden, dass erst mit biphasischen Schocks und dem Endotak™-System alle Patienten zuverlässig defibrillierbar waren [11]. Mit Jörg Neuzner als Erstautor einer Europäischen Multicenter-Studie unter Beteiligung von 15 deutschen Zentren bestätigte sich der Vorteil eines biphasischen ICD [12]. Die zuverlässige Defibrillation durch einen transvenösen ICD ohne benötigte Thorakotomie ermöglichte eine geringere Morbidität und Mortalität der ICD-Implantation und eine Durchführung der ICD-Implantation auch außerhalb herzchirurgischer Zentren. Die Implantationszahlen und -zentren wuchsen rasant. Um einen hohen Standard zu gewährleisten, kümmerten sich die ICD-Hersteller um umfassende Ausbildung. Die Trademarks AICD™ und PCD™ wurden zugunsten des generischen Kürzels „ICD“ aufgegeben. Die Firma CPI bzw. deren Nachfolgefirma Guidant, die den ICD weiter entwickelt hatte, betrieb von 1991–2002 ihr European Training Center in der Kerckhoff-Klinik Bad Nauheim (Martin Schlepper, Jörg Neuzner, Heinz F. Pitschner) mit Norbert Zanker als Manager [13]. Ab 2008 wurde durch die Deutsche Gesellschaft für Kardiologie (DGK) eine Sachkunde für ICD-Therapie vergeben, deren Anforderungen 2021 aktualisiert worden sind [14, 15].

**Figure 4:**
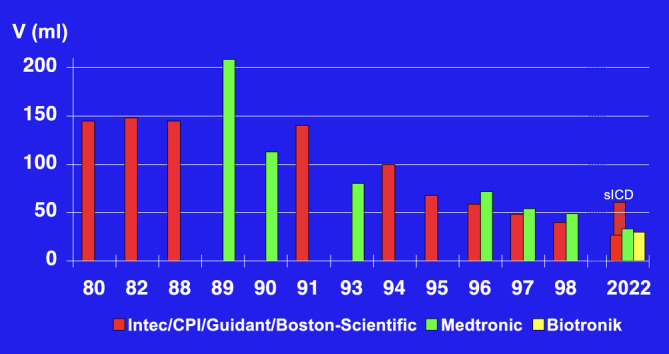

**Figure 5:**
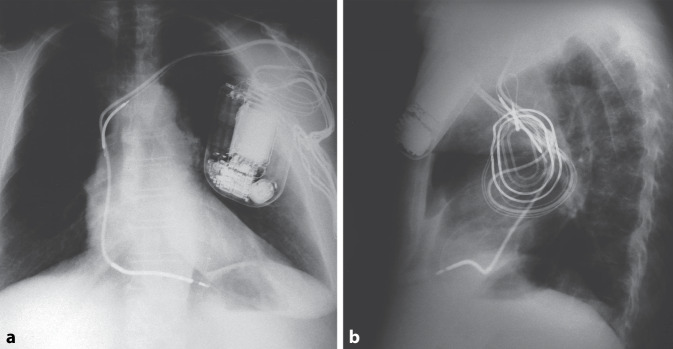

Eine weitere Verbesserung wurde durch die Verkleinerung des ICD-Aggregats und die damit mögliche pektorale anstatt Bauchwand-Implantation erreicht (Abb. 4). Weltweit wurde diese erstmals in Münster im Mai 1991 durchgeführt (Abb. 5; [16]). Seit ca. 1995 wurden alle Defibrillatoren pektoral implantiert. Damit konnte das Aggregat auch als Defibrillationselektrode genutzt und auf die subkutane Defibrillationselektrode verzichtet werden [17]. Der Wegbereiter für die „Active-can“-Technologie des ICD war Gust Bardy aus Seattle, der die nordamerikanisch/europäische Studie leitete (1993–1995), bei der Werner Jung aus Bonn für Europa der Principal Investigator war [18]. Nun bestand der transvenöse ICD standardmäßig aus einer rechtsventrikulären Single-coil-Elektrode und einem pektoral implantierten Aggregat. Jörg Neuzners Einsatz ist es zu verdanken, dass die bei Explantationen durch Verwachsungen in der oberen Hohlvene gefährliche Dual-coil-Variante der transvenösen Elektrode weitestgehend verlassen wurde [19]. Nachdem ein transvenöses ICD-System mit biphasischen Schocks äußerst zuverlässig defibrillierte, wurden die insbesondere bei schlechter linksventrikulärer Ejektionsfraktion gefährlichen intraoperativen Defibrillationstestungen immer häufiger nicht durchgeführt [20–22]. Dies bestätigten später zwei große randomisierte Studien (SIMPLE, NORDIC-ICD). SIMPLE, unter kanadischer Studienleitung, hatte 2 deutsche Mitautoren (Stefan Hohnloser und Jörg Neuzner; [23]), NORDIC-ICD lief als rein europäische Studie unter deutscher Studienleitung (Dietmar Bänsch) mit 7 deutschen Mitautoren [24].

## Entwicklung des subkutanen ICD (S-ICD) als Alternative sowie eines tragbaren Kardioverter-Defibrillators (WCD) als temporäre Alternative zum transvenösen ICD

Aufgrund der mit der Zeit immer deutlicher sichtbar werdenden Langzeit-Komplikationen der transvenösen Defibrillationselektroden [25, 26] wurde ein rein subkutanes Defibrillationssystem (S-ICD), erneut federführend von Gust Bardy aus Seattle und der Firma Cameron Health, San Clemente, California, USA (2012 aufgekauft durch Boston-Scientific Corporation, Marlborough, Massachusetts, USA), entwickelt, mit ersten Implantationen im Juli 2008 in Neuseeland [27]. An der zunächst intraoperativen Evaluierung eines subkutanen Defibrillationssystems nahmen auch die deutschen Kollegen Johann Sperzel, Jörg Neuzner und Stefan Spitzer teil [27] und an einer Studie zur Bestimmung der idealen Position der Schockelektroden für ein weiteres S‑ICD-System weitere deutsche Zentren mit Jürgen Kuschyk als Erstautor der Publikation [28]. Die erste Implantation eines S‑ICD in Deutschland erfolgte im Juni 2010 in Münster [29]. An der randomisierten Vergleichsstudie zum transvenösen ICD, die 2021 eine Nichtunterlegenheit des S‑ICD zeigte, nahmen mehrere deutsche Zentren teil (Mannheim, Kiel, München, Leipzig; [30]), auch an dem großen europäischen Register EFFORTLESS [31]. Es erfolgten zahlreiche deutsche Forschungsbeiträge, u. a. aus Düsseldorf zur Operationstechnik [32], aus Münster zur Zuverlässigkeit des Sensings [33] und aus Mannheim zur Kombination mit anderen implantierten Geräten wie Schrittmachern [34]. Einer der bekanntesten Experten auf dem Gebiet der S‑ICD-Technologie und Implantationstechnik wurde der Herzchirurg Joachim Winter aus Düsseldorf. In Erprobung befindet sich die Kombination mit einem Leadless Pacemaker, der mit dem S‑ICD kommuniziert und antitachykarde Stimulation ermöglicht [35]. Ein ähnlicher ICD der Firma Medtronic, als extravaskulärer ICD (EV-ICD) bezeichnet, ermöglichte durch eine substernale (statt subkutane) Elektrode antitachykardes Pacing (ATP) und für kurze Zeit Pacing zur Vermeidung von Pausen. In der Pivotal Study ohne deutsche Beteiligung von 2019 bis 2022 tolerierten bei hohem Pacing-Output nicht alle Patienten ATP [36].

Um Patienten in Phasen mit hohem Risiko für einen plötzlichen Herztod passager schützen zu können, wurde die tragbare Defibrillator-Weste (WCD) von der Firma LIFECOR, Pittsburgh, Pennsylvania, USA (später durch ZOLL Medical Corporation, Chelmsford, Massachusetts, USA aufgekauft) entwickelt. Der WCD überwacht wie ein ICD den Patienten, detektiert automatisch und zuverlässig ventrikuläre Tachyarrhythmien und kardiovertiert/defibrilliert mit programmierbaren Schocks. Solange der Patient bei Bewusstsein ist, kann er einen Knopf am in einer Tasche getragenen Monitor gedrückt halten, um die schmerzhafte Schockabgabe zu verhindern. Die Defibrillationselektroden befinden sich im Rückenteil der Weste und im anterolateralen Bereich des zur Weste gehörenden Gürtels. Auch die Sensing-Elektroden auf der Haut des Patienten befinden sich im Westengürtel. Der behandelnde Arzt kann zu jeder Zeit über einen Webserver der Firma Zoll das EKG und die Arrhythmie-Ereignisse des Patienten über das Internet erfragen. Auch wenn die Idee und technische Entwicklung aus den USA kamen (Stephen Heilman, Pittsburgh, der auch an der Entwicklung des ICD mit Michel Mirowski beteiligt war), wurden die ersten Studien in Deutschland an der Universität Magdeburg durchgeführt [37]. Zahlreiche prospektive Studien und Register zeigten die Sicherheit und Effektivität des WCD mit hoher Beteiligung deutscher Zentren, u. a. Magdeburg, Hannover, Dresden und Frankfurt [38–41]. An der randomisierten Studie zum Einsatz bei Patienten nach akutem Herzinfarkt (VEST-Trial) war Martin Borggrefe der einzige deutsche Koautor bei sechs teilnehmenden Studienzentren aus Deutschland [42]. Das primär negative Ergebnis der Studie (primärer Endpunkt war die Reduktion des plötzlichen Herztodes) ist durch die geringe tägliche Tragezeit des WCD erklärbar. Entsprechend zeigte die* On-treatment-Analyse* der Studie einen klaren Vorteil des WCD mit einer signifikanten Reduktion des plötzlichen Herztodes und der Gesamtmortalität [43].

## Integration der antitachykarden Stimulation und von Therapien für atriale Tachyarrhythmien in den ICD

Nur wenige Monate nach der Erstimplantation eines ICD in Deutschland wurde ein rein transvenöses System mit einem kleinen, subpektoral zu implantierenden Generator europaweit erstmals in Düsseldorf am 12.04.1984 implantiert. Dieses neue System hatte jedoch nur eine Schockenergie von 2 J, was zwar zur Kardioversion, nicht aber zur Defibrillation ausreichen würde. Angesichts nicht seltener Akzeleration der VT in ein Kammerflimmern war eine Energie von maximal 2 J unzureichend. Daher erwies sich diese Idee eines ausschließlichen Kardioverters ohne Defibrillationsmöglichkeit als Fehlweg [44]. Auch andere spezielle Schrittmacher mit ATP zur Terminierung von VT standen schon länger zur Verfügung, hatten aber auch das Problem nichtbeherrschbarer Akzelerationen zu Kammerflimmern. Im Vergleich zur Kardioversion war alleinige ATP für den Patienten schmerzlos; so lag es nahe, den ICD zusammen mit einem antitachykarden Schrittmacher zu implantieren. Schon 1986 wurden 5 Patienten mit einem System dieser Kombination durch die Universitätsklinik in Bonn versorgt [45]. Die Realisierung dieser Kombination in einem Gerät erfolgte 1989 im Pacer-Kardioverter-Defibrillator (PCD™) der Firma Medtronic. Zu dieser Zeit wurde die klinische Forschung der beiden amerikanischen Hersteller auf dem Gebiet der ICDs zum Teil von den USA nach Europa verschoben; erste klinischen Studien mit neuen ICDs fanden immer häufiger in Europa, vor allem in Deutschland statt. Bereits 1987 hatte Medtronic das Bakken Research Center in Maastricht gegründet. So wurde 1991 über die ersten multizentrischen Erfahrungen zur Effektivität des PCD™ in 11 europäischen Zentren, mit Beteiligung von 4 deutschen Zentren (Universitäten Hamburg, Heidelberg, München und Münster), zeitgleich mit den amerikanischen Erfahrungen berichtet [46]. In zahlreichen deutschen Zentren wurde zu ATP geforscht, so z. B. in Göttingen über Nichterfordernis einer Testung im elektrophysiologischen Labor [47], in Hannover zur Effektivität verschiedener ATP-Modi [48] sowie in Münster zum klinischen Einsatz bei hämodynamisch stabilen VT [49] und bei sehr schnellen VT [50]. Das Konzept der einmaligen ATP während der Kondensatoraufladung zur Defibrillation setzte sich bei den amerikanischen PainFree-Studien für schnelle VT durch [51, 52].

Am 03.06.1996 wurde erstmals in Bonn ein atrialer Defibrillator (METRIX Atrioverter, InControl Inc., Redmond, California, USA) zur Behandlung von Vorhofflimmern implantiert [53]. Trotz Nachweis von guter Effektivität und Sicherheit in einer Multicenterstudie unter Beteiligung von Bonn und Ludwigshafen [54] konnte sich dieses Therapieverfahren mit niedriger Schockenergie, aber immer noch schmerzhaften Schocks nicht durchsetzen – auch nicht bei Integration in einen Zweikammer-ICD (Medtronic Jewel™ AF), der auch fähig war, atriale Tachykardien und Vorhofflattern zu detektieren und mittels ATP oder Kardioversion über eine zusätzliche atriale Sense/Pace/Defibrillations-Elektrode zu terminieren [55]. Publiziert wurden die Ergebnisse der Multicenterstudie mit Beteiligung zahlreicher deutscher Zentren von Wolfgang Schoels.

## Vermeidung inadäquater Schocks

Nachdem die erste Generation der ICDs nur eine Abfrage darüber zuließ, wie viele Schocks insgesamt abgegeben worden waren, waren seit der 3. Generation der ICDs detaillierte gespeicherte Daten über therapierte Ereignisse in Form von Zykluslängen, Detektionsmarkern und Elektrogrammen abrufbar, bei Zweikammer-ICDs auch für den atrialen Kanal [12, 46]. Hierdurch wurde z. B. durch eine Analyse solcher Daten in einer prospektiven Studie durch das Adverse Event Committee mit deutscher Beteiligung aus Münster und Heidelberg erkannt, dass inadäquate Therapien ein Hauptproblem des ICDs waren [56]. Die ICDs der 3. Generation ermöglichten in Abhängigkeit von der Frequenz der ventrikulären Tachyarrhythmie eine individuelle Programmierung verschiedener Frequenzzonen mit unterschiedlichem Detektions- und Therapiemodus. Die verwendeten Detektionsalgorithmen wurden immer komplexer und unter Verwendung der Relation zwischen Vorhof- und Ventrikelsignal zum Teil zu einer „Blackbox“ für den Anwender. Zahlreiche Untersuchungen zur optimalen Programmierung der Tachykardie-Detektion wurden in deutschen Zentren durchgeführt, z. B. zur Differenzierung von VT und Sinustachykardien bzw. schnell auf die Kammer übergeleitetem Vorhofflimmern durch das *Sudden-onset-* bzw. *Stability-Kriterium* [57, 58] oder Vergleiche von Einkammer- zu Zweikammeralgorithmen [59–61]. Bis heute hat sich kein genereller Vorteil der Zweikammeralgorithmen gezeigt [62]. Die wichtigste randomisierte Studie zur Reduktion inadäquater Schocks kam aus den USA, hatte aber wie viele der MADIT-Publikationen, hier MADIT-RIT, Helmut Klein als deutschen Koautor [63]. Sie zeigte, dass eine Programmierung von Schocks erst ab einer Frequenz von 200/min aufwärts oder ATP/Schocks erst nach längerem Andauern (60 s) der VT bei Frequenzen zwischen 170/min und 199/min zu einer erheblichen Reduktion von inadäquaten Schocks ohne vermehrte Synkopen oder Todesfällen führte.

## Vermeidung adäquater Schocks

Neben der Terminierung von VT durch ATP und den Programmierungsvorschlägen aus MADIT-RIT zur Vermeidung einer unnötigen Schockbehandlung von VT konnte auch durch medikamentöse bzw. Katheterablationsbehandlung – insbesondere bei Patienten mit VT-Sturm – eine Verbesserung der Lebensqualität von ICD-Patienten erreicht werden. Sotalol konnte in einer doppelblinden, placebokontrollierten Studie eine deutliche Reduktion adäquater Schocks belegen. Neben vielen amerikanischen Studienzentren waren auch Frankfurt, Heidelberg und Münster beteiligt [64]. Beim Vergleich verschiedener antiarrhythmischer Medikamente zeigte in der größten randomisierten nordamerikanisch-europäischen OPTIC-Studie Amiodaron die beste Wirksamkeit. An dieser Studie beteiligten sich viele deutsche Zentren; Stefan Hohnloser war einer der Autoren [65]. Eine frühe deutsche Studie aus Münster (1993) zeigte die Wirksamkeit der Ablationsbehandlung von häufig auftretenden VT bei ICD-Patienten [66]. Bei der VT-Katheterablation ist eine von Karl-Heinz Kuck geleitete randomisierte Studie (VTACH) mit Beteiligung europäischer Zentren zu erwähnen, die vor einer ICD-Implantation bei ischämischer Kardiomyopathie und VT eine Ablation durchführte. Die Studie zeigte, dass VT-Rezidive nach einer Ablation verzögert auftreten [67]. Eine 2020 abgeschlossene Folgestudie (BERLIN VT) konnte aber nicht belegen, dass eine prophylaktische Ablation für die Mortalität einen Vorteil gegenüber einer erforderlichen Ablation im Verlauf der Nachbeobachtung hatte [68].

## Entwicklung der Leitlinien zur ICD-Indikation

Parallel zur kontinuierlichen Entwicklung der ICD-Therapie erfolgte die genaue Charakterisierung der Patienten, deren Leben durch den ICD verlängert wurde. Zunächst sollten nur Patienten mit einem ICD versorgt werden, die bereits anhaltende ventrikuläre Tachyarrhythmien hatten (sog. *Sekundärprävention*). Für dieses Patientenkollektiv wurden bereits 1991 Empfehlungen zur ICD-Implantation durch die North American Society of Pacing and Electrophysiology [69], das American College of Cardiology/American Heart Association [70] und die DGK [71] formuliert – und ein Jahr später auch von der European Society of Cardiology [72]. Zu dieser Zeit erfolgten fast alle Implantationen mittels Thorakotomie, und es gab noch keine randomisierten Studien. Der angenommene Überlebensvorteil durch den ICD basierte auf Überlebenskurven ohne adäquate effektive Schockabgaben oder Tod im Vergleich zu den tatsächlichen Überlebenskurven [73]. Es folgten drei randomisierte Studien mit Patienten der Sekundärpräventionsbehandlung (AVID, CIDS und CASH). Diese verglichen alleinige Antiarrhythmika-Therapie gegen ICD-Behandlung und wurden zwischen 1997 und 2000 publiziert. CASH war eine Hamburger Multicenterstudie unter Leitung von Karl-Heinz Kuck [74]. Eine Metaanalyse zeigte eine relative Reduktion der Mortalität von 27 % durch den ICD gegenüber Amiodaron und festigte damit die Indikationsstellung zur ICD-Sekundärprävention [75]. Bereits 1996 und 1997 waren zwei Studien zur Primärprophylaxe des plötzlichen Herztodes mittels ICD (MADIT, CABG-Patch) publiziert worden, an der als einzige nichtamerikanische Zentren Magdeburg und Hannover bzw. Münster und Heidelberg teilnahmen [76, 77]. Es folgten von 2002 bis 2009 sechs weitere randomisierte Studien zur Primärprophylaxe, mit zum Teil hohen Patientenzahlen (MADIT II, DINAMIT, SCDHeFT, IRIS, DEFINITE, AMIOVIRT). In allen Studien war eine hochgradig eingeschränkte linksventrikuläre Ejektionsfraktion das entscheidende Einschlusskriterium. Beide Postinfarktstudien (DINAMIT [78] und IRIS [79]) sowie eine Pilotstudie bei Patienten mit dilatativer Kardiomyopathie (CAT [80]) standen unter deutscher Leitung (Stefan Hohnloser, Gerhard Steinbeck, Karl-Heinz Kuck) mit der Beteiligung von vielen deutschen Implantationszentren. An der größten Studie für die ischämische Kardiomyopathie (MADIT II [81]) waren zwei deutschen Zentren (Magdeburg, Bad Nauheim) beteiligt. Zusammengefasst war die Botschaft der randomisierten Studien zur Primärprävention: Besteht trotz ausreichend langer Zeit nach kausalen Therapien und/oder Initiierung einer optimalen medikamentösen Therapie weiterhin eine hochgradig eingeschränkte linksventrikuläre Funktion, so ist ein ICD indiziert. Die Erkenntnisse für die Primärprävention bei seltenen Erkrankungen wurden und werden aus prospektiven Studien immer differenzierter – mit zahlreichen wissenschaftlichen Beiträgen aus Deutschland, z. B. zur Amyloidose [82], arrhythmogenen rechtsventrikulären Kardiomyopathie [83], hypertrophen obstruktiven Kardiomyopathie [84] und zum Short-QT-Syndrom [85]. Die letzten, rein ICD-bezogenen Leitlinien erschienen in Deutschland 2006 [86] und in den USA 2008 [87] mit einer Anpassung an neuere Daten 2012 [88]. Heute ist die Indikation zur ICD-Therapie in den Leitlinien zur Behandlung von VT bzw. Prävention des „Sudden Cardiac Death“, des „Heart Failure“ oder der „Cardiomyopathies“ integriert. Die Indikationen zur ICD-Therapie, sowohl zur Sekundär- wie auch zur Primärprävention blieben über die Jahre unverändert. Erst 2022 wurde die Indikation zur ICD-Primärprävention bei der nichtischämischen Kardiomyopathie wegen der 2016 publizierten DANISH-Studie zurückgestuft [89, 90]. Grund war die nicht mehr signifikante Reduktion der Mortalität durch die ICD-Therapie in dieser Studie. Seit 2015 sanken die ICD-Neuimplantationen in Deutschland von 30.002 auf 20.047 im Jahr 2021 um 33 % [91]. Die in Deutschland zurzeit laufende randomisierte RESET-CRT-Studie unter der Leitung von Gerhard Hindricks wird zeigen, ob bei Etablierung einer kardialen Resynchronisationstherapie (CRT), wie sie gehäuft in der DANISH Studie erfolgte, auch ein ICD benötigt wird (CRT-D) oder nicht (CRT-P) [92]. Die Ergebnisse eines sehr großen europäischen ICD-Registers (EU-CERT-ICD) zur primärprophylaktischen ICD-Behandlung bei ischämischer oder dilatativer Kardiomyopathie, mit hohen Patienten-Einschlussraten aus Deutschland, wurde 2021 von Markus Zabel (Göttingen) als Erstautor publiziert [93]. Sie zeigte für Patienten mit einer niedrigen linksventrikulären Ejektionsfraktion, aber ohne EKG-Kriterien für eine CRT-Indikation einen Überlebensvorteil von 27 %. Die Befunde dieser Untersuchung ergaben aber auch die Notwendigkeit einer besseren Individualisierung der ICD-Therapie. Ein dazu geeigneter Risikoscore wurde unter Beteiligung von Helmut Klein 2021 aus den MADIT-Studien kalkuliert [94]. Insbesondere die letzte ESC-Leitlinie zur Behandlung von Kardiomyopathien zeigt beispielhaft die zunehmende Individualisierung der ICD-Therapie in der Primärprävention auf, bei der neben der linksventrikulären Ejektionsfraktion zunehmend Genetik und Myokardvernarbung in der MRT sowie Risikoscores für bestimmte Kardiomyopathien zur Entscheidung beitragen [95].

## Beteiligung deutscher Firmen an der Entwicklung des ICD

Ein entscheidender Beitrag der Firma Siemens – über ihre schwedische Tochterfirma Elema in Stockholm – war nach der Implantation des weltweit ersten antibradykarden Schrittmachers (08.10.1958) die Entwicklung eines antitachykarden Schrittmachers (Prolog [96], Tachylog [97]). Siemens-Elema entwickelte auch einen ICD mit ATP. Die klinische Einführung dieses ICD scheiterte aber nach wenigen Implantationen an dem im Vergleich zu etablierten ICD-Herstellern sehr großen ICD-Aggregat, das nicht für eine pektorale Implantation geeignet war.

Die Firma Biotronik, Berlin, Deutschland, brachte erst 1993 ihren ersten ICD auf den Markt. Sie führte vor allem mit dem Prinzip des „Home Monitoring“ im Jahr 2000 ein System zur telemetrischen technischen ICD-Kontrolle und zur Nachsorge herzinsuffizienter Patienten ein, das jetzt alle ICD-Hersteller erfolgreich in ihre Geräte integriert haben. Randomisierte Multicenterstudien belegten die Vorteile des Home Monitoring [98, 99]. Zusätzlich führte Biotronik eine rechtsventrikuläre Defibrillationselektrode mit einem Sensing-Dipol im rechten Vorhof ein, um Zweikammer-Detektionsalgorithmen ohne eine zusätzliche atriale Pace-Sense-Elektrode verwenden zu können [100].
